# Platelet-rich plasma to treat ankle cartilage pathology - from translational potential to clinical evidence: a systematic review

**DOI:** 10.1186/s40634-015-0019-z

**Published:** 2015-02-12

**Authors:** Francesca Vannini, Berardo Di Matteo, Giuseppe Filardo

**Affiliations:** I Orthopaedic Clinic and Movement Analysis Laboratory, Rizzoli Orthopaedic Institute, Via di Barbiano n. 1/10, 40136 Bologna, Italy; II Orthopaedic Clinic and Biomechanics Laboratory, Rizzoli Orthopaedic Institute, Via di Barbiano n. 1/10, 40136 Bologna, Italy

**Keywords:** PRP, Growth factors, Ankle, Cartilage, Injections, Intra-articular

## Abstract

Platelet-rich Plasma (PRP) is a fascinating biological treatment showing promising results for the management of cartilage disorders. However, despite the step forwards in this research area and the increasing use of PRP in clinical practice, its use remains still controversial and especially its application as injective treatment for ankle cartilage pathology have been scarcely investigated.

The aim of this paper is to describe the translational evidence for the use of PRP in cartilage treatment and to systematically review all the available evidence regarding the clinical application of PRP for ankle cartilage disorders, in order to understand what is the current state of the art for this specific clinical indication, underlining both limits and potential of this biological strategy.

A systematic review of the clinical literature was performed on the use of PRP to treat ankle cartilage disorders and 7 papers were identified. PRP has been used in two different ways: 5 of the available papers focus on its use as an augmentation procedure to various surgical techniques for cartilage regeneration, while only two studies report its conservative application through intra-articular injections. Based on the limited number of clinical studies available on this topic, this systematic review showed the lack of major adverse events related to PRP and overall good results for the treatment of ankle cartilage pathology, thus confirming the translational potential of this biological treatment suggested by several preclinical studies. Further high quality clinical trials in the ankle are still needed to clarify proper indications and best applicative modalities.

## Introduction

Platelet-rich Plasma (PRP) is a fascinating biological treatment showing promise for the management of cartilage disorders [1,2]. The possibility of using autologous blood to obtain a product rich in growth factors and other bio-active molecules involved in tissue healing has been regarded as an innovative approach that could be sided to classical “on-the shelf” products from pharmaceutical companies.

The first reports in this field of application were published 10 years ago and since then many studies have been made in order to understand the biological effects of PRP and the potential and limits of this approach, not just as a therapeutic strategy for cartilage pathology [3,4] but more in general for a wide range of musculo-skeletal disorders, such as ligament and tendon pathologies [5-7]. However, despite the step forwards in this research area and the increasing use of PRP in the clinical practice, its use remains still controversial, especially its application as a treatment for ankle cartilage pathology [6].

In fact, under a clinical point of view, the vast majority of the studies already published is focused on the application of PRP in the knee joint, whereas only a smaller number of studies deals with other joints such as hip and ankle, either as a conservative injective approach or as an augmentation during surgical procedures for cartilage repair [8-11]. Thus, the rationale for the use of PRP in the ankle is currently mainly derived from the knee application. Among the available knee focused studies, 4 double blind randomized controlled trials show promising results and an overall support for the therapeutic potential of PRP [12-15]. PRP has been shown to provide a superior clinical outcome with respect to placebo (saline solution), thus proving a real clinical benefit [15]. More controversial is the superiority of the results obtained compared to other treatments: when compared to viscosupplementation, results are not that promising and there is some support but no conclusive evidence that PRP is significantly better than hyaluronic acid (HA) for the treatment of cartilage pathology [12]. The overall literature suggests that PRP is at least as effective as HA and should be therefore considered one of the available options for knee cartilage treatment. While knee pathology can be considered a bench test for all articular locations, other joints have been less enquired by researchers.

The aim of this paper is to describe the translational evidence for the use of PRP in cartilage treatment and to systematically review all the available evidence regarding the clinical application of PRP for ankle cartilage disorders, in order to understand what is the current state of the art for this specific clinical indication, underlining both limits and potential of this biological strategy.

## Review

### Materials and methods

**Figure 1:**
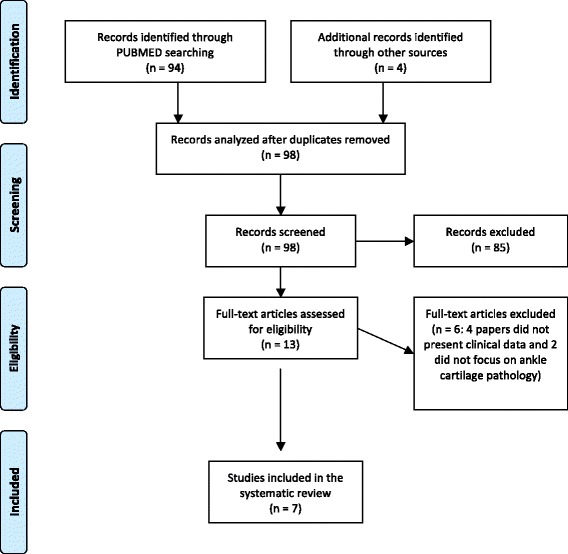
**Prisma 2009 flow diagram describing the papers selection process.**

A systematic review of the literature was performed on the use of PRP to treat ankle cartilage disorders. The search was made on the PubMed database on October 10th, 2014 using the following formula: (PRP OR platelet rich plasma OR platelet concentrate OR PRGF OR platelet gel OR platelet derived growth factors) AND (ankle or tibio-talar or talar) AND (osteoarthritis OR chondropathy OR chondral lesions OR osteochondral lesion). The guidelines for Preferred Reporting Items for Systematic Reviews and Meta-analysis (PRISMA) were used [16]. The screening process and analysis were performed separately by 2 independent researchers (Figure 1).

First, the articles were screened by title and abstract. The following inclusion criteria for relevant articles were used during the initial screening of titles and abstracts: clinical reports of any level of evidence, written in the English language, with no time limitation, on the use of PRP to treat ankle cartilage disorders. Other exclusion criteria were: case reports, articles written in other languages, animal trials, and reviews. In the second step, the full texts of the selected articles were screened, with further exclusions according to the previously described criteria. Moreover, articles not reporting clinical results were excluded. Reference lists from the selected papers were also screened. Relevant data were then extracted and collected in a single database with consensus of the two observers to be analyzed for the purposes of the present manuscript. At the end of the process, 7 papers fulfilled the selection criteria and have been described in the following paragraph.

## Results

PRP application, as reported in the literature for cartilage disease treatment in the ankle joint, can be summarized in two treatment strategies. Five papers focus on its use as an augmentation procedure to various surgical techniques for cartilage regeneration, while only two studies report its conservative application through intra-articular injections (Table 1).

**Table 1:** **Synopsis of all the clinical studies on PRP application in ankle cartilage pathology**

**AUTHORS, JOURNAL AND YEAR**	**LEVEL OF EVIDENCE**	**PATHOLOGY**	**PRP features**	**PROTOCOL**	**PATIENTS**	**FOLLOW- UP**	**OUTCOME**
Mei-Dan et al. AJSM 2012 [17]	I – Randomized trial	Osteochondral lesions of the talus	Leukocyte-poor PRP	3 injections of 2 ml PRP, at two-weeks interval	30 (15 PRP vs 15 HA)	7 months	Statistically significant better clinical outcome for PRP group
			Single centrifugation				
			Platelet count: 2-3x basal value				
			Activation: Ca-chloride				
Angthong et al. J Foot Ankle Surg 2013 [18]	IV – Case series	Ankle OA	Low platelet concentrate, leukocyte poor PRP	One injection of 3 ml PRP	5	16 months	Significant clinical improvement without any change in OA grade
			Single centrifugation				
			Activation: No				
Guney et al. KSTTA 2013 [19]	II – Randomized trial	Osteochondral lesion of the talus	Platelet count: 5.4x basal value	One PRP injection (6–24 hours after performing microfractures)	35 (19 microfractures + PRR vs 15 microfractures alone)	16 months	Combined treatment with PRP determined significantly better outcomes in terms of functional scores with respect to bone marrow stimulation alone.
			Leukocyte: n.a.				
			10% NAHCO3 addition before PRP injection				
Giannini et al. Clin Orthop Rel Res 2009 [20]	IV – case series	Osteochondral lesion of the talus	Double centrifugation, leukocyte rich PRF	PRP added intra-operatively to the biomaterial during the osteochondral lesion repair	48	24 months	Significant improvement in the AOFAS score of the patietns treated at final FU. 75% of the patients were able to resume sports activity. Cartilage with hyaline appearance in the 2 second look biopsies performed.
			Platelet count 5x basal value				
Giannini et al. Injury 2010 [21]	III – retrospective comparative trial	Osteochondral lesion of the talus		PRP added intra-operatively to the biomaterial during the osteochondral lesion repair	81 (25 one step technique with PRP vs 10 open ACI vs 46 arthroscopic ACI)	36 months	All the 3 groups had similar patterns of clinical improvement. The one step technique was cheaper.
Battaglia et al. Eur J Radiol 2011 [22]	IV – case series	Osteochondral lesion of the talus		PRP added intra-operatively to the biomaterial during the osteochondral lesion repair	20	24 months	Regenerated tissue with a T2 relaxation time value comparable to hyalinecartilage was found in all the treated cases, covering a mean of 78% of the repaired lesion area.
Giannini et al. Am J Sports med 2013 [23]	IV – case series	Osteochondral lesion of the talus		PRP added intra-operatively to the biomaterial during the osteochondral lesion repair	49	48 months	A significant relationship between the AOFAS score at 48 months’ follow-up and the percentage of regenerated cartilage with hyaline features (T2 value of 35–45 ms). Patients with hyaline-like regenerated cartilage in more than 80% of the treated area had a more predictable and stable result.

### Conservative application

Conservative application of PRP was firstly reported in a prospective randomized study by Mei-Dan et al. [17], who compared the efficacy of hyaluronic acid (HA) and PRP in 30 patients (15 per group) affected by talar osteochondral lesions not responsive to previous conservative management. Patients were divided into 2 groups: one to receive 3 weekly intra-articular injections of HA (2 ml each); the other one to receive PRP (2 ml each) with the same administration protocol. They were evaluated for up to 28 weeks of follow-up: AHFS (Ankle Hindfoot Score), AOFAS (American Orthopaedic Foot and Ankle Score), and VAS (Visual Analogue Scale) were used to test pain, stiffness and function, showing that PRP was significantly more effective at controlling pain and re-establishing function.

The conservative approach was also described by Angthong et al. [18], who applied PRP to different foot and ankle diseases and, among them, to low grades of ankle OA. PRP was injected under fluoroscope or ultrasound guidance and results were evaluated by VAS-FA (VAS Foot and Ankle) score. Patients with OA were also studied radiographically and scored for the OA grade. The 5 patients with OA included in this study presented a significant improvement in their clinical conditions according to VAS-FA score at a mean follow-up of 16 months (calculated on the whole series of patients). Despite the improved clinical condition, no changes in the degree of OA was evident on MRI 5 months after PRP injection.

### Surgical application

The surgical application of PRP as augmentation to microfractures in full thickness osteochondral lesions of the talus has been investigated by Guney et al. [19] in the only randomized comparative study available on this specific application modality: 35 patients were scheduled to receive either microfracture alone or microfracture in association with PRP (a single injection after 6–24 hours from arthroscopic microfractures), and were assessed at 16.2 (12–24) months follow-up using the AOFAS scoring system, Foot and Ankle Ability Measure (FAAM), and the VAS for pain. At baseline, AOFAS and FAAM scores were similar in the two groups, whereas pain scores (VAS) were higher in those who were assigned to the combined treatment. Despite the latter finding, the combined treatment with PRP resulted in significantly better outcomes in terms of functional scores (AOFAS, FAAM overall pain domain, FAAM 15-min walking domain, and pain-related scores) when compared to bone marrow stimulation alone. The authors concluded that PRP as an adjunct to arthroscopic microfracture surgery for the treatment of osteochondral lesions of the talus resulted in improved functional score status in the medium-term evaluation.

The most common surgical application of PRP involved its use as augmentation to scaffold implantation. This strategy for the treatment of full thickness talar osteochondral lesions has been documented by Giannini and his group in four different studies [20-23]. They described the clinical application of an arthroscopic one-stage technique involving autologous mesenchymal stem cells (MSCs), PRP, and either porcine collagen powder or HA membrane. The procedure consisted of harvesting bone-marrow-derived cells from the posterior iliac crest of the patients through a traditional marrow needle. Sixty ml of bone marrow-aspirate were collected and immediately put into a cell separator-concentrator to obtain a final 6-ml concentrate of MSCs. A collagen powder or a HA membrane could then be used. In the former cases, 2 ml of MSCs concentrate was added to 1 g of collagen powder and 1 ml of platelet-rich fibrin gel (obtained the day before surgery). In the latter cases, HA membrane was cut to match the size of the talar ostechondral lesion and then covered with 2 ml of MSCs concentrate and 1 ml of platelet-rich fibrin gel. The entire procedure was performed via ankle arthroscopy and, after the preparation of the lesion, the biological composite was placed in the defect site through a cannula and using a probe to obtain the best possible fit.

The first prospective single-arm clinical trial [20] involved 48 patients (mean age = 28.5 years) affected by focal lesions (mean size = 2.1 cm^2^) and evaluated at 6, 12, 18, and 24 months of follow-up using the AOFAS score. A significant increase in this parameter was recorded 6 months after the surgical procedure, and this outcome was confirmed up to the final follow-up. The rate of return to high impact sport activity was satisfactory: more than 75% of the patients returned to sport at 11 months of follow-up. The investigators found a correlation between clinical outcome and lesion size: poorer results were found for larger lesions, and previous surgery was also shown to affect the outcome negatively. Conversely, the outcome was not influenced by the lesion depth or by the type of scaffold used (collagen powder or HA membrane). Five second-look arthroscopies were performed at 1 year follow-up: in 2 cases biopsies were taken, revealing, after histologic and immune-histologic analysis, the presence of new cartilage tissue with varying degrees of tissue remodeling towards a hyaline aspect. The overall findings suggested that this novel approach could stimulate tissue regeneration with promising clinical efficacy, thus providing results that may even be comparable to those of autologous chondrocyte implantation (ACI) while avoiding the double surgical time and the inherent stress for the patient.

The same authors performed a further study [21] to compare MSCs + PRP + scaffold with open and arthroscopic ACI. Eighty-one patients were included in this analysis, and three different treatments were compared: 10 patients were treated by open ACI, 46 by arthroscopic ACI, and 25 by the bone-marrow-derived mesenchymal cells “one-step” technique. The clinical results were compared for up to 3 years of follow-up. AOFAS was the test chosen for clinical evaluation and radiographic analysis was performed as well. The clinical improvement in each subgroup was significant and no inter-group difference was observed, thus confirming the possibility of matching the effectiveness of chondrocyte transplantation by a single-step procedure. X-Rays showed no sign of OA progression and MRI revealed a good rate of defect filling and integration of the newly regenerated tissue within the surrounding tissue. Another aspect worth considering is the economic one: in fact, the authors pointed out that their novel one-step regenerative technique costs less than a half than traditional arthroscopic ACI.

An MRI evaluation was performed by Battaglia et al. [22] at 2 years’ follow-up from surgery in a selection of 20 patients operated by this one-step technique. MRI images were acquired using, beside a protocol suggested by the International Cartilage Repair Society and the MOCART scoring system, a T2 mapping qualitative sequence. Results were then correlated with AOFAS clinical score, which showed a significant improvement from pre-operatively to 2 years of follow-up. The T2 relaxation time value of 35–45 ms was derived from healthy ankles evaluation and assumed as normal hyaline cartilage value and used as a control. Regenerated tissue with a T2 relaxation time value comparable to hyaline cartilage was found in all the treated cases, covering a mean of 78% of the repaired lesion area. A high clinical score was directly correlated to iso-intense signal in DPFSE fat sat and to the percentage of regenerated hyaline cartilage, and inversely correlated to the percentage of newly formed fibrocartilage. Lesion’s depth was negatively correlated to the integrity of the repaired tissue’s surface and to the percentage of regenerated hyaline cartilage.

Finally, the same original case series of 49 patients was clinically evaluated at a further follow-up of 4 years [23]. The overall AOFAS score improved significantly, with best results at the 24-month follow-up. A significant decrease in the clinical score was observed between 24 and 36 months postoperatively and between 24 and 48 months in the general case series. Nevertheless, while evaluating the 20 patients who performed T2 mapping MRI 2 years before, it was evident a significant relationship between the AOFAS score at 48 months’ follow-up and the percentage of regenerated cartilage with hyaline features (T2 value of 35–45 ms). Patients with hyaline-like regenerated cartilage in more than 80% of the treated area had a more predictable and stable result.

## Discussion

The results of this systematic review show the lack of major adverse events related to PRP, and overall good results for the treatment of ankle cartilage pathology, either as conservative approach or as augmentation to surgical procedures, thus confirming the translational potential of this biological treatment suggested by several preclinical studies and also in this limited number of clinical trials regarding the ankle joint.

The literature exploring the potential of PRP in the preclinical setting underlined different aspects that could explain the positive clinical findings obtained with this blood derivative (Table 2).

**Table 2:** **Summary of the findings from in vitro and in vivo evidence concerning the application of PRP in cartilage pathology**

**IN VITRO EVIDENCE**		
**PRP EFFECTS ON…**	**Chondrocytes culture [** 3 **,** 25 **-** 27 **]**	- Increase chondrocyte proliferation rate
		- Stimulate chondrocyte phenotype expression and mainteinance
		- Increase matrix molecules production
		- Overall reduction of inflammatory response
	**Condrocytes + MSC culture [** 3 **,** 29 **]**	- Promotion of MSCs proliferation, adhesion and migration
		- Increased expression of Collagen II
	**Synovial Cells culture [** 3 **,** 28 **]**	- Anti or pro-flogistic effects demonstrated, according to the particular PRP formulation used
	**Meniscal cells culture [** 3 **,** 30 **]**	- Increase in meniscal cell proliferation rate
		- Stimulation of GAG synthesis
	**Stem Cells culture [** 3 **,** 24 **,** 29 **]**	- Stimulation of migration and chondrogenic differentiation of cortico-spongious bone cells
		- Superior chondrogenic differentiation of BMSCs
		- Enhanced proliferation rate and retained chondrogenic differentiation potential of ADMSCs
		- Increase in proliferation and migration induction of peripheral blood and umbilical cord blood MSCs
	**Bacterial culture [** 40 **]**	- PRP is able to provide an early protection against bacterial contamination due to the release of antimicrobial proteins
**ANIMAL MODELS**		
**PRP EFFECTS ON…**	**Rats [** 31 **]**	- In chondral lesion model: PRP improves tissue healing and collagen II expression
		- In OA model: controversial results regarding chondroprotective effects
	**Rabbit [** 32 **,** 33 **,** 36 **]**	- In Osteochondral lesion: no macroscopic, microscopic and biomechanical benefit after PRP administration
		- In OA model: positive influence on cartilage degeneration process (especially in moderate OA)
	**Sheep [** 35 **]**	- In chondral lesion model: improvement in macroscopic, histologic, and biomechanical cartilage scores. Beneficial effects also when applied together with microfractures
	**Pig [** 37 **]**	- In Rheumatoid Arthritis model: overall reduction in pro-flogistic enzymes and molecules; chondroprotective effect
	**Horse [** 34 **]**	- In OA model: positive modulation of joint homeostasis, with reduction of lameness and joint effusion up to middle term evaluation

*In vitro* studies investigating the effect of PRP on chondrocytes showed various and heterogeneous mechanisms of action [24-26]. Most of these studies agreed in reporting an increased chondrocyte proliferation rate, while the maintenance of chondrocyte marker expression has been observed in several papers but not unanimously confirmed. Other effects ranged from the matrix production stimulation to the inflammation modulation, and some findings even suggested a potential role of PRP as analgesic compound by modulating cannabinoid receptors in chondrocytes [27].

Beside the direct effects on chondrocytes, to understand the translational potential of PRP it is also important to consider the overall role played by PRP in affecting the entire joint homeostasis, as demonstrated by studies focused on different cell types. Platelet concentrates may significantly enhance synoviocyte HA secretion and switch synovial angiogenesis to a more balanced status, even though some reports also suggest some potential drawbacks, likely related to the leukocyte content, due to the metallo-proteinases production and therefore a possible pro-inflammatory response that could lead to an accelerated cartilage catabolism [28].

Since the overall PRP effect in the articular environment derives from an interaction with the pre-existing environment and other cells, and since some surgical protocols involve the application of both platelets and cells, several studies also investigated the effect of PRP on MSCs of different origin. In all cases an overall stimulatory effect was documented, ranging from a chemotactic action to an increased proliferation, and from the chondrogenic differentiation to the production of molecules specific of the articular cartilage [29,30].

Preclinical studies also explored the rational for PRP use and tested its translational potential in the animal model, with interesting findings in both an acute and chronic lesion setting. Induced OA was treated with PRP injections showing heterogeneous results in different experimental models: while some authors showed no significant effects in the rat model, others suggested an indirect effect through the induction of other cells and a final better histological appearance [29,31]. More concordant and favorable results have been seen in bigger animal models, with a suppression of OA progression or even tissue regeneration in rabbits [32,33], and a significant improvement of degree of lameness and joint effusion treating OA in horses [34].

The treatment of acute chondral or osteochondral lesions has also been documented [35,36]. In these experimental settings, PRP seemed to provide an overall beneficial effect when used as an injective approach or even when applied after microfracturing. The findings emerged from these animal studies showed that PRP was able to increase macroscopic, histologic and biomechanical scores, although a full restoration of hyaline cartilage has not been shown. Furthermore, PRP was also tested in the pig rheumatoid arthritis model [37], revealing an anti-inflammatory potential and a chondroprotective effect that would be worthy of more studies to understand the contribution of PRP as topical therapy even in this more complex immunological disease.

Beside the positive effects of this therapy demonstrated by most of the preclinical in vitro and animal studies, many questions remain still open and how to manage the numerous variables that could optimize PRP translational potential is still controversial.

In fact, the final outcome could be influenced by variables related to the product, such as dose, cell content, activation method and storage procedures, as well as application modality and concomitant procedures [38]. First of all, PRP dose could be of particular importance for the outcome since it is strictly linked to the amount of growth factors and bioactive molecules administered. Both *in vitro* and animal studies showed a time-dependent regulation and the dose-dependency effect of PRP, suggesting that a better clinical outcome can be achieved in function of the platelet count [26,39]. However, beside the amount of platelets, another and more debated aspect could play a major role for the intra-articular application of PRP: cell content, which is the most controversial aspect, especially with regard to the presence of leukocytes. In fact, even though leukocyte presence could contribute to an anti-microbial effect [40], some in vitro studies have shown that leukocyte rich PRP stimulates expression of pro-inflammatory molecules and enzymes detrimental for joint homeostasis [41]. Nonetheless, from a clinical point of view there is a lack of studies addressing this question with a proper methodology and, looking at the current evidence, it has not been demonstrated any significant clinical difference according to PRP formulation, with the exception of post-injective adverse events (more pain and swelling after leukocyte-rich PRP administration) [42]. Activation methods and storage procedures are also aspects under examination: in particular, activation has been demonstrated to affect the physical properties and growth factors release kinetics of PRP [43], even if no data have been reported about differences in clinical results. For what regards storage methods, a recently published *in vitro* trial revealed that freeze thawing is a safe procedure, which sufficiently preserves PRP quality and its ability to induce proliferation and the production of extra cellular matrix (ECM) components in both chondrocytes and synoviocytes [44]. The possibility to preserve PRP could be relevant in the clinical setting since multiple injections of PRP can be required in case of poor response or symptom relapse, and this strategy is therefore attractive: a single blood harvesting can contribute to reduce costs and patient’s discomfort. However, therapeutic protocols (i.e.: number of PRP injections and their timing) are still a controversial point, where poor evidence is available, and clinicians tend to adopt very personal approaches, mainly based to their personal experience with other injective products [38].

In conclusion, the translational evidence currently available is overall supporting the application of PRP as a treatment option for both focal chondral/osteochondral lesion and for joint degenerative pathology, due to the milieau of effects exerted by PRP on all the articular tissues. For sure the great variability among PRP formulations, reported by in vitro studies, still remains the main issue to address in order to develop the best product for specific clinical use.

Looking at the available evidence on the clinical benefit obtained with this blood derivative, the application of PRP to treat ankle cartilage pathology is supported by 7 studies: 5 of them dealing with surgical treatment and two with an injective approach [17-23]. The randomized trial by Mei-Dan et al. [17] revealed significant results in favor of PRP but the low number of patients and the short-term follow-up evaluation are weak points, while the study by Angthong et al. [18] deals with the use of PRP in different foot and ankle pathologies and just 5 patients were actually affected by ankle OA. Conversely, the studies testing the surgical application of MSCs + PRP + biomaterials (collagen powder or HA membrane) [19-23] are well documented but the value in determining the actual role of PRP is limited because in all of them it is described the combined use of multiple biological autologous and bio-engineered substances, without control groups, so that it is impossible to determine the actual contribution of PRP itself to the success of the procedure [9].

## Conclusion

PRP is certainly a fascinating area of pre-clinical and clinical research. Nonetheless, at present, there is a very limited number of studies regarding the use of PRP for ankle cartilage pathology, since only one Level I trial is available and the surgical application, although particularly successful, relies in the majority of the cases on a combination of different factors.

Many “shadow lines” must be addressed: PRP cannot still be univocally defined and, up to now, there are too many different PRP formulations applied in clinical practice. The many inter-products differences previously discussed and related to preparation methods, cell content, activation and storage procedures, therapeutic protocols and others, reflect the complexity of this biological treatment and justify the difficulties in data comparison among studies and the inherent lack of clear indications about PRP therapy. Despite these limitations, this variability has stimulated a large interest of basic scientists and clinicians to explore what are the best characteristics of PRP to treat specific diseases, and many studies are ongoing to further improve this biological treatment approach. However, regardless of the golden aura around its therapeutic potential, until further studies will prove the real translational potential of the promising results suggested by preclinical *in vitro* and animal studies, thus optimizing its use and identifying the best indications in the clinical practice, PRP should be considered a second line treatment and applied in humans within well controlled studies.

## References

[1] Boswell SG, Cole BJ, Sundman EA, Karas V, Fortier LA (2012). Platelet-rich plasma: a milieu of bioactive factors. Arthroscopy.

[2] Kon E, Filardo G, Di Martino A, Marcacci M (2011). Platelet-rich plasma (PRP) to treat sports injuries: evidence to support its use. Knee Surg Sports Traumatol Arthrosc.

[3] Filardo G, Kon E, Roffi A, Di Matteo B, Merli ML, Marcacci M (2013). Platelet-rich plasma: why intra-articular? A systematic review of preclinical studies and clinical evidence on PRP for joint degeneration. Knee Surg Sports Traumatol Arthrosc. doi:10.1007/s00167-013-2743-1 10.1007/s00167-013-2743-1 PMC4541701 24275957

[4] Kon E, Filardo G, Di Matteo B, Marcacci M (2013). PRP for the treatment of cartilage pathology. Open Orthop J.

[5] Di Matteo B, Filardo G, Kon E, Marcacci M (2014). Platelet-rich plasma: evidence for the treatment of patellar and Achilles tendinopathy-a systematic review. Musculoskelet Surg. doi:10.1007/s12306-014-0340-1 10.1007/s12306-014-0340-1 25323041

[6] Filardo G, Kon E, Di Matteo B, Pelotti P, Di Martino A, Marcacci M (2013). Platelet-rich plasma for the treatment of patellar tendinopathy: clinical and imaging findings at medium-term follow-up. Int Orthop.

[7] Filardo G, Presti ML, Kon E, Marcacci M (2010). Nonoperative biological treatment approach for partial Achilles tendon lesion. Orthopedics.

[8] Filardo G, Kon E, Di Matteo B, Di Martino A, Tesei G, Pelotti P, Cenacchi A, Marcacci M (2014). Platelet-rich plasma injections for the treatment of refractory Achilles tendinopathy: results at 4 years. Blood Transfus.

[9] Vannini F, Di Matteo B, Filardo G, Kon E, Marcacci M, Giannini S (2014). Platelet-rich plasma for foot and ankle pathologies: a systematic review. Foot Ankle Surg.

[10] Battaglia M, Guaraldi F, Vannini F, Rossi G, Timoncini A, Buda R, Giannini S (2013). Efficacy of ultrasound-guided intra-articular injections of platelet-rich plasma versus hyaluronic acid for hip osteoarthritis. Orthopedics.

[11] Battaglia M, Guaraldi F, Vannini F, Buscio T, Buda R, Galletti S, Giannini S (2011). Platelet-rich plasma (PRP) intra-articular ultrasound-guided injections as a possible treatment for hip osteoarthritis: a pilot study. Clin Exp Rheumatol.

[12] Filardo G, Kon E, Di Martino A, Di Matteo B, Merli ML, Cenacchi A, Fornasari PM, Marcacci M (2012). Platelet-rich plasma vs hyaluronic acid to treat knee degenerative pathology: study design and preliminary results of a randomized controlled trial. BMC Musculoskelet Disord.

[13] Sánchez M, Fiz N, Azofra J, Usabiaga J, Aduriz Recalde E, Garcia Gutierrez A, Albillos J, Gárate R, Aguirre JJ, Padilla S, Orive G, Anitua E (2012). A randomized clinical trial evaluating plasma rich in growth factors (PRGF-Endoret) versus hyaluronic acid in the short-term treatment of symptomatic knee osteoarthritis. Arthroscopy.

[14] Cerza F, Carnì S, Carcangiu A, Di Vavo I, Schiavilla V, Pecora A, De Biasi G, Ciuffreda M (2012). Comparison between hyaluronic acid and platelet-rich plasma, intra-articular infiltration in the treatment of gonarthrosis. Am J Sports Med.

[15] Patel S, Dhillon MS, Aggarwal S, Marwaha N, Jain A (2013). Treatment with platelet-rich plasma is more effective than placebo for knee osteoarthritis: a prospective, double-blind, randomized trial. Am J Sports Med.

[16] Moher D, Liberati A, Tetzlaff J, Altman DG, The PRISMA Group (2009). Preferred reporting items for systematic reviews and meta-analyses: the PRISMA statement. Open Med.

[17] Mei-Dan O, Carmont MR, Laver L, Mann G, Maffulli N, Nyska M (2012). Platelet-rich plasma or hyaluronate in the management of osteochondral lesions of the talus. Am J Sports Med.

[18] Angthong C1, Khadsongkram A, Angthong W (2013). Outcomes and quality of life after platelet-rich plasma therapy in patients with recalcitrant hindfoot and ankle diseases: a preliminary report of 12 patients. J Foot Ankle Surg.

[19] Guney A, Akar M, Karaman I, Oner M, Guney B (2013). Clinical outcomes of platelet rich plasma (PRP) as an adjunct to microfracture surgery in osteochondral lesions of the talus. Knee Surg Sports Traumatol Arthrosc. doi:10.1007/s00167-013-2784-5 10.1007/s00167-013-2784-5 24292979

[20] Giannini S, Buda R, Vannini F, Cavallo M, Grigolo B (2009). One-step bone marrow-derived cell transplantation in talar osteochondral lesions. Clin Orthop Relat Res.

[21] Giannini S, Buda R, Cavallo M, Ruffilli A, Cenacchi A, Cavallo C, Vannini F (2010). Cartilage repair evolution in post-traumatic osteochondral lesions of the talus: from open field autologous chondrocyte to bone-marrow-derived cells transplantation. Injury.

[22] Battaglia M, Rimondi E, Monti C, Guaraldi F, Sant'Andrea A, Buda R, Cavallo M, Giannini S, Vannini F (2011). Validity of T2 mapping in characterization of the regeneration tissue by bone marrow derived cell transplantation in osteochondral lesions of the ankle. Eur J Radiol.

[23] Giannini S, Buda R, Battaglia M, Cavallo M, Ruffilli A, Ramponi L, Pagliazzi G, Vannini F (2013). One-step repair in talar osteochondral lesions: 4-year clinical results and t2-mapping capability in outcome prediction. Am J Sports Med.

[24] Hildner F, Eder MJ, Hofer K, Aberl J, Redl H, van Griensven M, Gabriel C, Peterbauer-Scherb A (2013). Human platelet lysate successfully promotes proliferation and subsequent chondrogenic differentiation of adipose-derived stem cells: a comparison with articular chondrocytes. J Tissue Eng Regen Med. doi:10.1002/term.1649 10.1002/term.1649 23303715

[25] Muraglia A, Ottonello C, Spanò R, Dozin B, Strada P, Grandizio M, Cancedda R, Mastrogiacomo M (2013). Biological activity of a standardized freeze-dried platelet derivative to be used as cell culture medium supplement. Platelets. doi:10.3109/09537104.2013.803529 10.3109/09537104.2013.803529 23885791

[26] Park SI, Lee HR, Kim S, Ahn MW, Do SH (2012). Time-sequential modulation in expression of growth factors from platelet-rich plasma (PRP) on the chondrocyte cultures. Mol Cell Biochem.

[27] Lee HR, Park KM, Joung YK, Park KD, Do SH (2012). Platelet- rich plasma loaded hydrogel scaffold enhances chondrogenic differentiation and maturation with up-regulation of CB1 and CB2. J Control Release.

[28] Assirelli E, Filardo G, Mariani E, Kon E, Roffi A, Vaccaro F, Marcacci M, Facchini A, Pulsatelli L (2014). Effect of two different preparations of platelet-rich plasma on synoviocytes. Knee Surg Sports Traumatol Arthrosc. doi:10.1007/s00167-014-3113-3 10.1007/s00167-014-3113-3 PMC4541703 24942296

[29] Mifune Y, Matsumoto T, Takayama K, Ota S, Li H, MeszarosLB UA, Nagamune K, Gharaibeh B, Fu FH, Huard J (2013). The effect of platelet-rich plasma on the regenerative therapy of muscle derived stem cells for articular cartilage repair. Osteoarthritis Cartil.

[30] Moreira Teixeira LS, Leijten JC, Wennink JW, Chatterjea AG, Feijen J, van Blitterswijk CA, Dijkstra PJ, Karperien M (2012). The effect of platelet lysate supplementation of a dextran-based hydrogel on cartilage formation. Biomaterials.

[31] Guner S, Buyukbebeci O (2012). Analyzing the effects of platelet gel on knee osteoarthritis in the rat model. Clin Appl Thromb Hemost. doi:10.1177/1076029612452117 10.1177/1076029612452117 22790657

[32] Kwon DR, Park GY, Lee SU (2012). The effects of intra-articular platelet-rich plasma injection according to the severity of collagenase-induced knee osteoarthritis in a rabbit model. Ann Rehabil Med.

[33] Saito M, Takahashi KA, Arai Y, Inoue A, Sakao K, Tonomura H, Honjo K, Nakagawa S, Inoue H, Tabata Y, Kubo T (2009). Intraarticular administration of platelet-rich plasma with biodegradable gelatin hydrogel microspheres prevents osteoarthritis progression in the rabbit knee. Clin Exp Rheumatol.

[34] Carmona JU, Arguelles D, Climent F, Prades M (2007). Autologous platelet concentrates as a treatment of horses with osteoarthritis: a preliminary pilot clinical study. J Equin Vet Sci.

[35] Milano G, Deriu L, Sanna Passino E, Masala G, Saccomanno MF, Postacchini R, Fabbriciani C (2011). The effect of autologous conditioned plasma on the treatment of focal chondral defects of the knee. An experimental study. Int J Immunopathol Pharmacol.

[36] Serra CI, Soler C, Carillo JM, Sopena JJ, Redondo JI, Cugat R (2013). Effect of autologous platelet-rich plasma on the repair of full-thickness articular defects in rabbits. Knee Surg Sports Traumatol Arthrosc.

[37] Lippross S, Moeller B, Haas H, Tohidnezhad M, Steubesand N, Wruck CJ, Kurz B, Seekamp A, Pufe T, Varoga D (2011). Intraarticular injection of platelet-rich plasma reduces inflammation in a pig model of rheumatoid arthritis of the knee joint. Arthritis Rheum.

[38] Tschon M, Fini M, Giardino R, Filardo G, Dallari D, Torricelli P, Martini L, Giavaresi G, Kon E, Maltarello MC, Nicolini A, Carpi A (2011). Lights and shadows concerning platelet products for musculoskeletal regeneration. Front Biosci (Elite Ed).

[39] Torricelli P, Fini M, Filardo G, Tschon M, Pischedda M, Pacorini A, Kon E, Giardino R (2011). Regenerative medicine for the treatment of musculoskeletal overuse injuries in competition horses. Int Orthop.

[40] Mariani E, Filardo G, Canella V, Berlingeri A, Bielli A, Cattini L, Landini MP, Kon E, Marcacci M, Facchini A (2014). Platelet-rich plasma affects bacterial growth in vitro. Cytotherapy.

[41] Cavallo C, Filardo G, Mariani E, Kon E, Marcacci M, Pereira Ruiz MT, Facchini A, Grigolo B (2014). Comparison of platelet-rich plasma formulations for cartilage healing: an in vitro study. J Bone Joint Surg Am. 5;96 (5):423–429. 10.2106/JBJS.M.00726 24599205

[42] Filardo G, Kon E, Pereira Ruiz MT, Vaccaro F, Guitaldi R, Di Martino A, Cenacchi A, Fornasari PM, Marcacci M (2012). Platelet-rich plasma intra-articular injections for cartilage degeneration and osteoarthritis: single- versus double-spinning approach. Knee Surg Sports Traumatol Arthrosc.

[43] Dohan Ehrenfest DM, Bielecki T, Jimbo R, Barbé G, Del Corso M, Inchingolo F, Sammartino G (2012). Do the fibrin architecture and leukocyte content influence the growth factor release of platelet concentrates? An evidence-based answer comparing a pure platelet-rich plasma (P-PRP) gel and a leukocyte- and platelet-rich fibrin (L-PRF). Curr Pharm Biotechnol.

[44] Roffi A, Filardo G, Assirelli E, Cavallo C, Cenacchi A, Facchini A, Grigolo B, Kon E, Mariani E, Pratelli L, Pulsatelli L, Marcacci M (2014). Does platelet-rich plasma freeze-thawing influence growth factor release and their effects on chondrocytes and synoviocytes? Biomed Res Int. doi:10.1155/2014/692913 10.1155/2014/692913 PMC4124719 25136613

